# Supplementation of feed and water after long-duration road transportation: The effects on welfare and rumen fermentation in goats

**DOI:** 10.3389/fvets.2023.1135666

**Published:** 2023-03-28

**Authors:** Ke Xu, Kang Yang, Yi Yang, Wenxuan Wu, Chuanshe Zhou

**Affiliations:** ^1^Key Lab of Animal Genetics, Breeding and Reproduction in the Plateau Mountainous Region, Ministry of Education, Guizhou University, Guiyang, China; ^2^Department of Animal Science, Institute of Animal Nutrition and Feed Science, Animal Science College, Guizhou University, Guiyang, China; ^3^Institute of New Rural Development, Guizhou University, Guiyang, China; ^4^Key Lab of Agro-Ecological Processes in Subtropical Region, Institute of Subtropical Agriculture, Chinese Academy of Sciences, Changsha, China

**Keywords:** road transportation stress, nutrients supplementation, plasma metabolites, rumen fermentation, goats

## Abstract

Water and feed are needed for livestock during their long-duration road transportation. However, limited information is available on the need to supply water and feed to livestock at temporary holding stations after road transportation. This study aimed to evaluate the effect of providing water and feed at holding stations on the welfare of goats in mimic surroundings. A total of 24 Guizhou black goats were randomly divided into three groups of eight goats each as follows: deprived of water and feed (TRT0), supplemented with water *ad libitum* (TRT1), and supplemented with water and feed *ad libitum* (TRT2). Blood and rumen fluid samples were collected before loading (denoted as “PRE” in this article) and after transport (denoted as “POST” in this article). Statistical analysis was performed *via* the SAS procedure PROC MIXED. The 10-h road transportation period reduced body weight in TRT0 goats (*p* < 0.05) but not in TRT1 and TRT2 (*p* > 0.05). TRT0 and TRT1 goats had POST plasma glucose concentrations above their PRE values (*p* < 0.05). The PRE-plasma urea nitrogen (PUN) levels were higher in TRT2 compared to TRT0 (*p* < 0.05) goats, while the POST–PUN levels increased in TRT1 compared to TRT0 goats. The POST non-esterified fatty acid (NEFA) concentration was higher for TRT0 compared to that in TRT1 and TRT2 (*p* < 0.05) goats. No difference was observed for plasma profiles of malondialdehyde (MDA) and superoxide dismutase (SOD) (*p* > 0.05). TRT2 goats had higher POST glutathione peroxidase (GSH-Px) activity than TRT0 and TRT1 (*p* < 0.05) goats. TRT0 goats had higher POST plasma glucagon (GC) compared to TRT2 (*p* < 0.05) and had increased values compared to their own PRE level as a result of road transportation (*p* < 0.05). TRT2 goats resulted in a lower POST plasma heat-stressed protein-70 (HSP-70) level than TRT0. There was no difference in ruminal pH (*p* > 0.05). Ruminal total VFA (acetate, propionate, butyrate), and the NH_3_-ammonia profiles showed a decrease (*p* < 0.05) after transportation in all groups. Ruminal microcrystalline cellulose, xylanase, cellobiase, and carboxymethyl cellulose activities were unaffected (*p* > 0.05). These combined results imply that water and feed supplementation to livestock can effectively alleviate stress responses in goats subjected to road transportation and emphasize the necessity to establish water and feed supplies even at a temporary holding pen.

## 1. Introduction

Goat meat has been consumed worldwide throughout human history, and it is commonly consumed in Southwest China during the winter. During the 3-month period from November to January, ~1 million goats are transported by road from rural areas to larger towns or major cities in China. To meet this demand, goats from different breeding farms are delivered to abattoirs for direct butchering or to finishing farms near cities for short-term fattening.

The typical duration of road transportation from the farm to unloading at a temporary holding pen is around 10 h. The inevitable practice of road transportation has been attracting increasing attention (1, 2) because it can result in body weight loss (3, 4); an increase in plasma catecholamines and cortisol levels (5); the promotion of blood corticotropin-releasing hormone, adrenocorticotropic hormone (ACTH), and glucocorticoid (GC) secretion (6, 7); and affect gut microbial counts and meat quality (8, 9). Ultimately, these disorders reduce both animal welfare and profits made on the animals. Another critical feature that results from the withdrawal of feed and water during road transportation of goats is reduced ruminal function. During transport, limiting ruminal nutrient input can affect rumen microbiota populations, leading to a reduction in volatile fatty acids (VFA) concentrations (10). Furthermore, stress *per se* during transport has been shown to disrupt rumen microbial fermentation and nutrient digestibility. Thus, feed and water restrictions after road transportation may further aggravate ruminal function and affect health status.

As the significance of alleviating stress during road transportation is widely acknowledged, several pretransport methods (11–13) have been evaluated. However, to the best of the authors' knowledge, the effects of feed and water supplementation on livestock after unloading have not been studied yet. We hypothesized that goats would recover more quickly if they were supplemented with feed and water after road transportation and conducted a mimic experiment to examine this hypothesis. More precisely, we examined whether water and feed supplementation in the cattle influence the growth performance, plasma metabolites of biochemical parameters, antioxidant capacity, stress markers, and rumen fermentation characteristics of goats. In addition, this study serves as an important theoretical supplement to previous research.

## 2. Materials and methods

### 2.1. Ethics approval

All experimental procedures were approved by the ethics committee of Guizhou University.

### 2.2. Animal management and experimental design

Twenty-four Guizhou male black goats (a local breed; 10 months of age and a mean of 25.04 [±1.41] kg body weight) in good health and free from clinical disease were used in this study. The goats were randomly divided into three groups of eight animals each as follows: deprived of water and feed all the time (TRT0, *n* = 8), supplemented with water *ad libitum* (TRT1, *n* = 8), and supplemented with feed and water *ad libitum* (TRT2, *n* = 8), respectively. Transport was carried out during the typical season of mutton consumption in December 2020 with an air temperature range from −1.6 to 5.9°C. Before the experiment started, all goats were given free access to water. On the experimental day, a truck was used to transport goats with a maximum loading weight of 4 t. The truck was open with no climate control. A straw and wood shavings bedding was provided to prevent the goats from slipping. The transportation compartment of the truck measured 3.3 m (length) × 1.7 m (width) × 2.2 m (height). For rapid identification, each goat was marked and numbered on both flanks with blue, green, or red dyes for TRT0, TRT1, and TRT2 goats, respectively.

The road journey started at 14:00 h and lasted 10 h; it covered a mountainous distance of 400 km, with ~75% of the journey on village roads and 25% on state highways. The driving speed was maintained at close to 40 km/h to prevent crowding. No feed or water was provided during transportation. After transportation and unloading, the goats were kept in separate cages measured at 1.5 m (length) × 1.2 m (width) × 1.5 m (height); and then, they were subjected to their respective treatments to stimulate temporary holding station conditions. TRT0 goats continued to be deprived of feed and water, TRT1 goats were supplemented with water *ad libitum* alone, and TRT2 goats were supplemented with feed and water *ad libitum*. The diet included alfalfa hay (30 mm chop length) and a pellet with a 40:60 ratio of concentrate to roughage (20 × 6 mm). The composition (%) of the concentrate is 61% of corn, 15% of soybean meal, 8% of rapeseed meal, 11% of wheat bran, 0.5% of CaHPO_4_, 0.2% of lysine, 0.3% of methionine, 1% of NaCl, and 3% of premix. Water provided to goats was warmed by pouring boiled water into the tap water, and the temperature was about 20°C. Goat behavior was observed through the transparent glass of the cages. Body weight was measured before road transportation (PRE) and after water and feed supplementation after road transportation (POST).

### 2.3. Sample collection and analysis

Blood samples were taken from all goats *via* jugular venipuncture. After the completion of road transportation, the goats were allowed 20 min to rest before the POST sampling. The samples were collected using a disposable vacuum tube with heparin for plasma. The plasma was stored at −20°C for later analysis. Plasma samples were analyzed for glucose (GLU), urea nitrogen (PUN), glutamic pyruvate transaminase (GPT), and non-esterified fatty acid (NEFA); malondialdehyde (MDA), superoxide dismutase (SOD), and glutathione peroxidase (GSH-PX), adrenocorticotropic hormone (ACTH), glucagon (GC), heat stress protein-70 (HSP-70), triiodothyronine (T3), and thyroxine (T4). All analyses were performed using commercially available kits (Nanjing Jiancheng Institute of Biological Engineering Company, China).

After blood sampling, a total of 5 goats in each group (a total of 15 goats for 3 groups) were randomly chosen for the collection of rumen fluid *via* an oral tubular collector. The initial stream of 100 ml of rumen fluid was discarded to prevent contamination with reticulum fluid or saliva. The next 200 ml of rumen fluid was collected for subsequent analysis. Rumen fluid pH was measured immediately (Seven2GoTM, Mettler Toledo). Aliquots of 150-ml of rumen fluid were stratified and then centrifugated at 15,000*g* for 20 min to collect the supernatant for the analysis of VFA concentrations using a chromatograph (Shimadzu GC-9A, Japan). Further 50-ml aliquots were stratified for the determination of rumen ammonia nitrogen (14) and cellulase activity (15).

### 2.4. Data statistical analysis

All statistical analyses were performed using SAS 9.4 (SAS Institute Inc., Cary, NC, USA), and all data were presented as means ± SD. The linear mixed model (PROC MIXED) was used to analyze nutrient supplementation treatment, transportation treatment, and transportation treatment × nutrient supplementation interactions. The auto regression (AR) (1) covariance structure was selected based on the lower Bayesian information criterion (BIC) and Akaike information criterion (AIC) values. Multiple comparisons among means were determined using Duncan's adjustment. The significance level was set as a *p*-value of < 0.05.

During the analysis of the rumen fermentation parameters for the five goats in TRT0, unfortunately, it was found that all the goats' rumen fluid was abnormally thick; therefore, analyses could not be reliable ones; thus, the samples were discarded and no further measurements were made on them. Consequently, rumen fermentation parameters were simply analyzed between TRT1 and TRT2.

## 3. Results

During road transportation, all goats struggled to maintain their balance. Under general observation, goats displayed symptoms of vocalization, vibration, kneeling, urination, defecation, sniffling, salivation, and trembling during road transportation.

### 3.1. Body weight

A significant road transportation (*p* < 0.05) effect was observed on body weight (Table 1). The POST body weight was decreased compared to PRE values in all three groups; goats in TRT0 had a significant body weight loss after road transportation (Δ = −1.87 kg), while TRT1 and TRT2 only showed a numerically decreased body weight loss (Δ = −1.63 and −0.62 kg, respectively).

**Table 1:** Effect of nutrient supplementation on live body weight variation in goats before and after transportation.

**Trans**.	**Supple**.			***p*** **-value**		
	**TRT0**	**TRT1**	**TRT2**	**Supple**.	**Trans**.	**Supple**. × **Trans**.
PRE	25.32 ± 1.11^a^	25.23 ± 1.53	24.58 ± 1.62	0.952	<0.05	0.766
POST	23.45 ± 1.39^b^	23.60 ± 1.99	23.96 ± 1.73			

Data were given as mean ± standard deviation (SD).

a,b Values with different superscript letters in the same column indicate a difference of transportation treatment (*p* < 0.05).

Trans., transportation treatment; Supple., nutrients supplementation treatment; TRT0, neither water nor feed supplementation; TRT1, water supplementation alone; TRT2, water and feed supplementation; PRE, before transportation, POST, after transportation.

### 3.2. Plasma biochemical parameters

Plasma biochemical parameters are shown in Table 2. A significant road transportation effect (*p* < 0.05) was observed for plasma GLU concentration. A higher POST plasma GLU concentration was observed for TRT0 and TRT1 goats compared to their PRE values (*p* < 0.05) but was not observed for TRT2 goats (*p* > 0.05). A significant nutrient supplementation treatment effect (*p* < 0.05) was observed in plasma PUN and NEFA concentrations. The PRE PUN concentrations were significantly higher (*p* < 0.05) in TRT2 goats compared to TRT0 goats, while TRT1 goats showed increased POST PUN levels (*p* < 0.05). The POST NEFA concentration was higher in TRT0 compared to TRT1 and TRT2 (*p* < 0.05) goats. There were no significant differences in nutrient supplementation treatment, road transportation, or their interaction for plasma profiles of GPT.

**Table 2:** Effect of nutrients supplementation on plasma biochemical metabolites in goats before and after transportation.

	**Trans**.	**Supple**.			***p*** **-value**		
		**TRT0**	**TRT1**	**TRT2**	**Supple**.	**Trans**.	**Supple**. × **Trans**.
GLU	PRE	3.97 ± 0.32^b^	4.33 ± 0.86^b^	3.99 ± 0.37	0.41	<0.05	0.43
	POST	7.36 ± 1.54^a^	6.34 ± 2.17^a^	5.30 ± 1.13			
PUN	PRE	3.48 ± 0.59^B^	4.40 ± 0.90^AB^	5.15 ± 1.29^A^	<0.05	0.87	0.43
	POST	3.05 ± 0.98^B^	4.96 ± 0.95^A^	4.12 ± 1.61^AB^			
NEFA	PRE	36.73 ± 17.46	35.87 ± 24.63	23.21 ± 6.14	<0.05	0.25	0.89
	POST	44.54 ± 11.66^A^	29.60 ± 10.96^B^	25.41 ± 6.76^B^			
GPT	PRE	16.53 ± 3.05	28.68 ± 12.78	24.96 ± 12.50	0.53	0.80	0.47
	POST	23.54 ± 3.92	21.92 ± 4.79	22.76 ± 6.29			

Data were given as mean ± standard deviation (SD).

a,b Values with different superscript letters in the same column indicate a difference of transportation treatment (*p* < 0.05).

A,B Values with different superscript uppercase letters in the same row indicate a difference for supplementation treatment (*p* < 0.05).

Trans., transportation treatment; Supple., nutrients supplementation treatment; TRT0, neither water nor feed supplementation; TRT1, water supplementation; TRT2, water and feed supplementation; PRE, before transportation; POST, after transportation.

### 3.3. Plasma antioxidant capacity

Plasma antioxidant capacity is listed in Table 3. There was an increasing trend for POST plasma MDA level (*p* = 0.06), in which TRT0 and TRT2 goats showed the highest and the lowest concentrations, respectively. No significant nutrient supplementation treatment, road transportation, or their interaction was observed for plasma profiles of SOD. A significant nutrient supplementation treatment effect (*p* < 0.05) was observed in plasma GSH-Px activity, and the POST activity was higher for TRT2 than for the other two groups.

**Table 3:** Effect of nutrient supplementation on plasma antioxidant measurements in goats before and after transportation.

	**Trans**.	**Supple**.			***p*** **-value**		
		**TRT0**	**TRT1**	**TRT2**	**Supple**.	**Trans**.	**Supple**. × **Trans**.
MDA	PRE	1.92 ± 0.94	1.58 ± 0.66	2.12 ± 0.75	0.19	0.06	0.16
	POST	4.35 ± 2.24	2.75 ± 1.38	2.54 ± 1.31			
SOD	PRE	20.43 ± 1.12	19.98 ± 1.42	20.44 ± 1.07	0.16	0.53	0.59
	POST	17.36 ± 2.88	20.47 ± 2.46	20.27 ± 1.03			
GSH-Px	PRE	369.79 ± 31.22	362.61 ± 48.09	377.62 ± 29.01	<0.05	0.22	0.31
	POST	341.50 ± 54.78^B^	329.62 ± 31.59^B^	383.87 ± 28.08^A^			

Data were given as mean ± standard deviation (SD).

^a,b^Values with different superscript letters in the same column indicate a difference for transportation treatment (*p* < 0.05).

A,B Values with different superscript uppercase letters in the same row indicate a difference for supplementation treatment (*p* < 0.05).

Trans., transportation treatment; Supple., nutrients supplementation treatment; TRT0, neither water nor feed supplementation; TRT1, water supplementation; TRT2, water and feed supplementation; PRE, before transportation; POST, after transportation.

### 3.4. Plasma stress markers

Plasma stress markers levels are shown in Table 4. A trend of the road transportation effect was only observed for plasma concentration of ACTH (*p* = 0.10), in which TRT2 goats had the lowest plasma ACTH level. TRT0 goats had higher (*p* < 0.05) POST plasma GC relative to TRT2 goats in response to the nutrient supplementation treatment and had increased (*p* < 0.05) values compared to their corresponding PRE level as a result of road transportation. A significant effect was observed in the plasma concentration of HSP-70 (*p* < 0.05), and TRT2 goats showed the lowest plasma HSP-70 level. The levels of two plasma thyroid hormones (T3 and T4) were unaffected by nutrient supplementation treatment, road transportation, or their interaction.

**Table 4:** Effect of nutrient supplementation on plasma stress status in goats before and after transportation.

	**Trans**.	**Supple**.			***p*** **-value**		
		**TRT0**	**TRT1**	**TRT2**	**Supple**.	**Trans**.	**Supple**. × **Trans**.
ACTH	PRE	40.25 ± 14.07	31.27 ± 12.13	25.46 ± 16.92	0.10	0.89	0.55
	POST	51.27 ± 15.46	30.03 ± 11.38	23.99 ± 6.10			
GC	PRE	39.12 ± 17.93^b^	29.87 ± 13.01	29.32 ± 13.30	<0.05	<0.05	0.42
	POST	47.36 ± 18.52^A^^a^	30.76 ± 11.81^AB^	24.59 ± 4.94^B^			
HSP-70	PRE	5.92 ± 2.14	5.41 ± 3.51	4.41 ± 1.27	<0.05	0.81	0.20
	POST	7.73 ± 2.67^A^	4.99 ± 2.85^AB^	3.09 ± 1.82^B^			
T3	PRE	341.08 ± 104.62	414.47 ± 142.25	312.14 ± 181.10	0.56	0.28	0.94
	POST	368.37 ± 115.31	408.72 ± 183.20	282.14 ± 35.71			
T4	PRE	4.60 ± 2.28	4.02 ± 1.55	3.47 ± 1.41	0.97	0.54	0.83
	POST	4.96 ± 1.71	4.54 ± 1.98	3.72 ± 0.84			

Data were given as mean ± standard deviation (SD).

a,b Values with different superscript letters in the same column indicate a difference for transportation treatment (*p* < 0.05).

A,B Values with different superscript upper case letters in the same row indicate a difference for supplementation treatment (*p* < 0.05).

Trans., transportation treatment; Supple., nutrients supplementation treatment; TRT0, neither water nor feed supplementation; TRT1, water supplementation; TRT2, water and feed supplementation; PRE, before transportation; POST, after transportation.

### 3.5. Rumen fermentation parameters and cellulase activity

Rumen fermentation parameters and cellulase activity are presented in Table 5. Ruminal pH was unaffected by nutrient supplementation treatment, road transportation, or their interaction. Ruminal total VFA (acetate, propionate, and butyrate) and NH_3_-ammonia profiles showed a significant decrease (*p* < 0.05) after transport in all groups. Furthermore, TRT2 goats had numerically greater total VFA, acetate, propionate, butyrate, and NH_3_-ammonia profiles than TRT1 goats. No differences were observed in ruminal microcrystalline cellulose, xylanase, cellobiase, and carboxymethyl cellulose activities for goats based on nutrient supplementation treatment, road transportation, or their interaction (*p* > 0.05).

**Table 5:** Effect of nutrient supplementation on ruminal fermentation parameters and cellulase activity in goats before and after transportation.

	**Trans**.	**Supple**.		***p*** **-value**		
		**TRT1**	**TRT2**	**Supple**.	**Trans**.	**Supple**. × **Trans**.
pH	PRE	6.63 ± 0.10	6.58 ± 0.14	>0.05	>0.05	>0.05
	POST	6.60 ± 0.12	6.52 ± 0.17			
TVFA	PRE	70.92 ± 6.10^a^	64.76 ± 4.70^a^	0.23	<0.05	0.18
	POST	32.26 ± 4.67^b^	45.17 ± 5.24^b^			
Acetate	PRE	44.33 ± 4.21^a^	36.08 ± 6.15^a^	0.52	<0.05	0.13
	POST	19.07 ± 3.96^b^	27.69 ± 4.55^b^			
Propionate	PRE	19.25 ± 2.16^a^	20.89 ± 1.80^a^	0.19	<0.05	0.94
	POST	9.79 ± 0.59^b^	12.97 ± 1.50^b^			
Butyrate	PRE	7.34 ± 0.54^a^	7.79 ± 1.43^a^	0.29	<0.05	0.67
	POST	3.40 ± 0.47^b^	4.52 ± 1.28^b^			
NH_3_-ammonia	PRE	17.00 ± 2.70^a^	18.43 ± 1.85^a^	0.14	<0.05	0.28
	POST	9.88 ± 2.52^b^	11.81 ± 1.35^b^			
Microcrystalline cellulose	PRE	2.36 ± 0.33	2.66 ± 0.35	0.93	0.50	0.12
	POST	2.56 ± 0.25	2.20 ± 0.15			
Xylanase	PRE	6.21 ± 1.41	7.41 ± 0.64	0.45	0.27	0.64
	POST	6.55 ± 0.93	6.63 ± 0.65			
Cellobiase	PRE	2.92 ± 0.12	3.26 ± 0.55	0.53	0.69	0.66
	POST	3.23 ± 0.19	3.22 ± 0.10			
Carboxymethyl cellulose	PRE	3.03 ± 0.17	3.12 ± 0.30	0.91	0.12	0.71
	POST	3.33 ± 0.17	3.29 ± 0.10			

Data were given as mean ± standard deviation (SD).

a,b Values with different superscript letters in the same column indicate a difference for transportation treatment (*p* < 0.05).

^A,B^Values with different superscript uppercase letters in the same row indicate a difference for supplementation treatment (*p* < 0.05).

Trans., transportation treatment; Supple., nutrients supplementation treatment; TRT1, water supplementation; TRT2, water and diet supplementation; PRE, before transportation; POST, after transportation.

## 4. Discussion

Transportation is a complex stressor made up of many factors including fluctuating temperatures, stocking density, withdrawal from feed and water, mixing with unfamiliar animals, and motion. These factors have the potential to activate the hypothalamic–pituitary–adrenal axis (HPA) (6, 7); therefore, transportation has the potential to affect the health and welfare of animals. However, the role of water and feed supplementation in road-transported goats is still undetermined. In this study, we aimed to evaluate the effects of water and feed supplementation in road-transported goats. The results showed that nutrient-supplemented goats had a superior recovery status.

### 4.1. Effect of nutrient supplementation on body weight in goats before and after transportation

Animals commonly lose body weight after transportation, especially if they have no access to feed and water (16, 17). Researchers report that road transportation causes a reduction in the live weight of small ruminants (3, 18, 19). This may be attributed to transportation-induced behavior, including urination, defecation, crowding, salivation, and probably gastrointestinal tract emptying. Our results indicated that 10 h of road transportation led to a reduction in body weight even though the goats were supplemented with water and feed. TRT0, TRT1, and TRT2 goats had a loss of body weight of 1.87, 1.63, and 0.62 kg after road transportation, respectively, accounting for a shrinkage of 7.38, 6.46, and 2.52%. Moreover, both TRT0 and TRT1 goats displayed a 2.92- and 2.56-fold body weight loss, respectively, in comparison to TRT2, indicating that the most severe dehydration occurred both in TRT0 and in TRT1 goats. This result suggested that feed supplementation, irrespective of water supplementation, played a crucial role in minimizing body weight loss in road-transported goats. To date, there have been few behavioral studies on road-transported animals; more studies are needed to quantify the relationship between body weight loss and stress behaviors.

### 4.2. Effect of nutrient supplementation on plasma biochemical metabolites in goats before and after transportation

The results indicated that both TRT0 and TRT1 goats had higher POST plasma GLU levels than their PRE values. This was probably owing to glycogenolysis, the breakdown of liver glycogen, or the depletion of skeletal muscle glycogen reserves that occurred in TRT0 and TRT1. A similar finding is reported by previous studies (20, 21). Hence, we speculated that plasma GLU concentration might be a valuable indicator of stress in road-transported goats. This phenomenon was not present in TRT2, implying that the TRT2 treatment was more effective in easing activation of the adrenal medulla, regulating plasma GLU homeostasis, and reducing road transportation stress. PUN is produced by protein breakdown and amino acid metabolism, and its concentration depends on crude protein levels (22). In our study, road transportation did not affect PUN concentration, implying no significant protein breakdown. This finding agrees with a previous study, which found no change in PUN concentration in donkeys after road transportation (23).

The NEFA is a good marker of body fat utilization and is often secreted into the blood. In the present study, both TRT1 and TRT2 goats^*^ had lower plasma NEFA levels compared to TRT0 goats, implying that nutrient supplementation may have reduced the extent of body fat mobilization. This is consistent with previous research, which found that road transportation greatly increases plasma NEFA concentration in animals (24). No interactive effects of road transportation and nutrient supplementation were found for the above-mentioned plasma biochemical metabolites. Few studies to date have evaluated plasma GTP when animals undergo road transportation. This study showed no difference in PRE and POST plasma GTP; the reason for these results needs further investigation.

### 4.3. Effect of nutrient supplementation on plasma antioxidant capacity in goats before and after transportation

Increased reactive oxygen species, owing to road transportation, may be responsible for oxidative stress in animals (25). Plasma MDA concentration is used as a common biomarker for assessing lipid peroxidation in biological and medical sciences (26). In the present study, a decreasing trend (*p* = 0.06) in plasma MDA concentration was observed after road transportation. This tend is in line with previous studies (4, 27, 28) and might be because of free-radical-induced lipid peroxidation of erythrocyte membranes. Furthermore, it was interesting to note that plasma SOD was unaffected by road transportation. This phenomenon also existed in our later, large-scale study with Guizhou black goats (*n* = 42), in which the goats were found to have similar plasma SOD levels before and after road transportation (personal data, not published). This discrepancy may be because the goats used in the study were a native breed. In parallel, TRT2 had significantly increased POST plasma GSH-Px levels relative to the other two groups. An increased GSH-Px level would serve as an oxygen radical scavenger to alleviate oxidative stress (25); thus, it may be speculated that water and feed supplementation eased the lipid peroxidation caused by road transportation stress.

### 4.4. Effects of nutrient supplementation on plasma stress in goats before and after transportation

We found that TRT2 had lower POST plasma ACTH (*p* = 0.10) and GC concentrations compared to TRT0 goats, suggesting that road transportation stress increased thyroid and adrenal functions, indicating that water and feed supplementation can alleviate road transportation stress in goats. Fazio et al. (29) reported an increase in blood ACTH and GC concentrations in cattle after road transportation (29).

Heat shock proteins (HSPs) are a group of evolutionarily conserved proteins synthesized in response to stressors (30), including transport stress. Thermal-induced stress enhances HSP synthesis, thus protecting stressed cells, preventing aggregation, or reversing disorders (31, 32). The HSP-70 levels in the kidney and the liver of the animals were also significantly increased by road transportation (21). Consistent with changes in plasma ACTH and GC concentrations, TRT2 goats showed lower POST plasma HSP-70 concentrations in this study. This significant reduction in crucial stress indicators suggests the utility of water and feed supplementation to mitigate stress in goats subjected to road transportation.

The levels of plasma T3 and T4 were increased in response to elevated glucose in road-transported goats (33), implying an increased activity of the HPA axis. However, plasma T3 and T4 levels were unchanged by road transportation or nutrient supplementation in the current study. This could be because the goats used in this study were a local mountainous breed that is highly adaptable.

### 4.5. Effect of nutrient supplementation on rumen fermentation parameters and cellulose activity in goats before and after transportation

Rumen pH is crucial for ruminants and is related to many factors, especially dietary constituents. The commonly reported level of rumen pH ranges from 6.0 to 8.0. In this study, rumen pH was similar both in TRT1 and TRT2 before and after road transportation. An increase in rumen pH had been noted in steers that experienced a 32-h of fast and transportation (34); however, the rumen pH values was in the normal range. We found that, after road transportation, all goats had significantly reduced rumen levels of TVFA (acetate, propionate, and butyrate) and NH_3_-ammonia, in agreement with a previous study (34). This finding suggests that road transportation exerted a dramatic influence on rumen fermentation.

We had expected that TRT2 goats would have more POST rumen cellulose activity because they were given additional feed rather than water alone, as in the case of TRT1 goats. It was surprising that rumen microcrystalline cellulose, xylanase, cellobiase, and carboxymethyl cellulose activities were unaffected, especially when the values were similar. These findings were in contrast to a previous study (10) which reported that ruminal microbiota was changed after road transportation and that cattle and yaks had higher levels of Firmicutes and Lactobacillus than Simmental crossbred cattle and native yellow cattle. There is no clear explanation for this result to date, and the absence of sampling and data collection on TRT0 further limited our analyses. To the best of our knowledge, there are insufficient studies investigating the effects of nutrient supplementation on rumen fermentation parameters for road-transported goats; thus, more research is needed. However, the lack of blank control, namely, not being transported and kept in the farm compared to the transported goats limits our deeper discussion in the current manuscript and needs further study. Overall, our result may be a useful way and be recommended in the new regulations to reduce the transportation stress and then warrant animals' welfare.

## 5. Conclusion

It is a known fact that road transportation may induce a physiological stress response and shift rumen fermentation, thereby leading to live weight loss in goats after transportation. The above results showed that water and feed supplementation can effectively alleviate the stress response and improve the health status of goats subjected to road transportation. Therefore, new supplementation strategies should be introduced, even at temporary holding stations, to enhance goat health and behavior.

## Data availability statement

The original contributions presented in the study are included in the article/supplementary material, further inquiries can be directed to the corresponding author.

## Ethics statement

The animal study was reviewed and approved by the experiment procedures were approved by the Ethics Committee of the Guizhou University. Written informed consent was obtained from the owners for the participation of their animals in this study.

## Author contributions

KX: conceptualization, investigation, sampling, formal analysis, data collection, visualization, and writing the original draft. KY: data curation, sampling, software, and editing the drafts. YY: experimental design, methodology, and data statistics. WW: project administration, supervision, and review of the drafts. CZ: resources, funding acquisition, and project administration. All authors have read and agreed to the published version of the manuscript.
